# Root Form and Root Canal Configuration of Permanent Maxillary First Molars in the Meerut Population: An In Vivo Cone-Beam Computed Tomography Study

**DOI:** 10.7759/cureus.106940

**Published:** 2026-04-13

**Authors:** Duddu Sravanteja, Panna Mangat, Viswesh Subramani, Mampi Biswas, Nayan Shree, Anmol Prajapati, Seema Gupta

**Affiliations:** 1 Department of Conservative Dentistry and Endodontics, Kalka Dental College, Meerut, IND; 2 Department of Orthodontics, Kothiwal Dental College and Research Centre, Moradabad, IND

**Keywords:** cone-beam computed tomography, maxillary first molar, mesiobuccal, prospective, root morphology

## Abstract

Background

Endodontic success in maxillary first molars depends on accurate knowledge of root and canal morphology, particularly the frequently complex mesiobuccal (MB) root, which often harbors an additional MB2 canal. Population-specific variations necessitate region-focused studies using advanced imaging techniques such as cone-beam computed tomography (CBCT). The aim of this in vivo CBCT study was to evaluate the root form and root canal configuration of permanent maxillary first molars in the Meerut population, with the primary objective of determining the prevalence of the MB2 canal and accessory canals in other roots and to classify morphologies using both the Vertucci and Ahmed systems. The secondary objectives included assessing sex-based differences and generating region-specific anatomical data to enhance preoperative planning and treatment outcomes.

Methodology

This prospective observational study included 300 permanent maxillary first molars from CBCT scans of individuals aged 18-70 years acquired for various clinical indications. The scans were evaluated using multiplanar reconstruction (voxel size ≤0.2 mm). Root number, canal number, and configuration were classified according to Vertucci’s system; non-fitting morphologies were further described using Ahmed’s (orifice-canal-foramen (O-C-F) coding. Inter and intraobserver reliabilities were confirmed (κ > 0.85). Data were analyzed descriptively using inferential statistics (chi-square, Cohen’s kappa, and correspondence analysis, with p-values <0.05 considered significant).

Results

A total of 300 participants (300 maxillary first molars) were included in the study. The MB root showed the highest anatomical complexity, with approximately 180 (60%) cases demonstrating MB2-related configurations, predominantly Vertucci Types II and IV. In contrast, the distobuccal and palatal roots exhibited simple canal morphology, with over 282 (>94%) cases presenting Type I configurations. Ahmed’s classification system successfully categorized all 300 (100%) cases, including 15 (5%) MB roots that were unclassifiable under Vertucci’s system, and showed almost perfect to perfect agreement (κ = 0.96-1.00). No statistically significant differences were observed between male and female participants in terms of root or canal configuration distribution.

Conclusions

The MB root of the maxillary first molars in the Meerut population displayed substantial anatomical variability, with a high prevalence of MB2 canals. The Ahmed classification offered superior descriptive details and eliminated unclassifiable cases compared to the Vertucci classification. These findings underscore the need for heightened vigilance, preoperative CBCT evaluation when indicated, and enhanced detection techniques to improve endodontic predictability in northern Indian populations.

## Introduction

Successful endodontic treatment of multirooted posterior teeth, particularly the permanent maxillary first molar, relies heavily on a thorough understanding of root and root canal morphology [1]. The maxillary first molar is among the most frequently treated posterior teeth owing to its early eruption, heavy occlusal load, susceptibility to caries, and restorative procedures. Its internal anatomy is notoriously complex, especially in the mesiobuccal (MB) root, which frequently harbors an additional canal commonly referred to as the second mesiobuccal (MB2) canal [2,3]. Failure to locate, negotiate, clean, shape, and obturate all existing canals remains one of the leading causes of endodontic treatment failure, with untreated canals frequently implicated in persistent periapical pathology [4].

Root canal morphology exhibits significant variation and is influenced by genetic, ethnic, geographic, and environmental factors. Classic studies using clearing techniques have established foundational classifications, most notably Vertucci’s eight-type system (1984), which describes patterns of canal division and merger from the pulp chamber to the apical foramen [5]. However, limitations of traditional classifications, particularly their inability to fully describe complex, multi-rooted morphologies, accessory canals, and intricate merging patterns, have prompted the development of more comprehensive systems that provide detailed orifice-canal-foramen (O-C-F) coding [5].

Cone-beam computed tomography (CBCT) has revolutionized noninvasive, three-dimensional evaluation of root canal anatomy, offering superior diagnostic accuracy compared to conventional periapical radiography by eliminating superimposition and enabling precise visualization in the axial, coronal, and sagittal planes [6]. Numerous CBCT-based studies across global populations have reported an MB2 prevalence ranging from approximately 50% to 70% in maxillary first molars, with notable ethnic and regional differences [7]. In Indian subpopulations, previous studies have indicated an overall MB2 prevalence of approximately 60-65%, although values vary widely depending on geographic location, age group, and imaging parameters [8,9].

Despite extensive research, region-specific data remain limited, particularly for northern Indian populations, such as Meerut (Uttar Pradesh), where diverse ethnic and migratory influences may contribute to unique anatomical patterns. Population-specific knowledge is clinically essential for guiding access cavity design, anticipating additional canals, selecting appropriate magnification and ultrasonic troughing techniques, and minimizing procedural errors, such as missed canals, perforation, or excessive dentin removal.

This in vivo study aimed to evaluate the root form and root canal configuration of permanent maxillary first molars in the Meerut population using CBCT. The primary objectives were to determine the prevalence of the MB2 canal and any accessory canals in the distobuccal and palatal roots, as well as to classify the root canal morphology of the mesiobuccal, distobuccal, and palatal roots according to different classification systems. The secondary objectives included assessing any sex-based differences in the observed root and canal configurations and generating region-specific anatomical data to support more accurate preoperative planning, improved canal detection, prevention of procedural errors (such as missed canals, perforation, or excessive dentin removal), and enhanced predictability of endodontic treatment outcomes in the local population.

## Materials and methods

This prospective observational cohort study was conducted at the Department of Conservative Dentistry and Endodontics, Kalka Dental College and Hospital, Meerut, Uttar Pradesh, India. Data acquisition was conducted continuously from May 2024 to December 2025. The study protocol was approved by the Institutional Ethics Committee of Kalka Dental College (IEC approval number: KDC/LTR/2024/097, dated April 25, 2024) and was conducted in accordance with the ethical principles of the Declaration of Helsinki (2013 revision). Written informed consent, provided in either English or Hindi according to participant preference, was obtained from each individual before inclusion. The participants were fully informed that participation was voluntary, that they could withdraw at any time without any impact on their routine dental care, and that all personal data would remain confidential.

Participants were eligible if they were between 18 and 70 years of age, had at least one permanent maxillary first molar (FDI teeth 16 or 26) with fully formed roots, and had undergone a CBCT scan of diagnostic quality for legitimate clinical indications, such as implant site evaluation, orthodontic planning, maxillofacial trauma assessment, or routine radiographic examination.

Individuals were excluded if they were younger than 18 years or older than 70 years, if the maxillary first molar under evaluation exhibited fused roots, if there was evidence of internal or external root resorption, if the tooth presented open apices indicating incomplete root maturation, if deep caries extended to the furcation area compromising reliable canal assessment, or if severe calcification obstructed clear visualization of the root canal system on CBCT images.

The required sample size was calculated using the G*Power software (version 3.1.9.7; Heinrich-Heine-Universität Düsseldorf, Germany). An effect size of 0.4 was considered based on the expected prevalence of complex root canal morphology in maxillary molars. This study was designed with a statistical power of 80% and an alpha error of 5%. The minimum required sample size was 280 CBCT scans. To account for potential exclusions due to poor image quality or incomplete data, the sample size was increased to 300 CBCT scans to ensure adequate power and reliability of the study results.

Each participant contributed only one tooth to the study to avoid clustering effects and ensure statistical independence of observations. In cases where both maxillary first molars (teeth 16 and 26) were present and met the inclusion criteria, one tooth was selected using a simple randomization approach. The recruitment flow involved screening CBCT records obtained for routine clinical indications, assessing eligibility based on predefined inclusion and exclusion criteria, and consecutively including qualifying cases until the required sample size of 300 was achieved.

CBCT scans were acquired using standard dental CBCT units with a field of view of 8 × 8 cm or 5 × 5 cm, encompassing the maxillary arch, tube voltage of 90-120 kV, tube current of 5-10 mA, and isotropic voxel size ≤0.2 mm. The exported DICOM datasets were evaluated on a high-resolution monitor under controlled ambient lighting using CS 3D Imaging Lite software (Carestream Dental LLC, Atlanta, Georgia, USA). Multiplanar reconstruction (axial, coronal, and sagittal planes) was performed with a uniform slice thickness of 250 µm to ensure consistent visualization.

**Figure 1:**
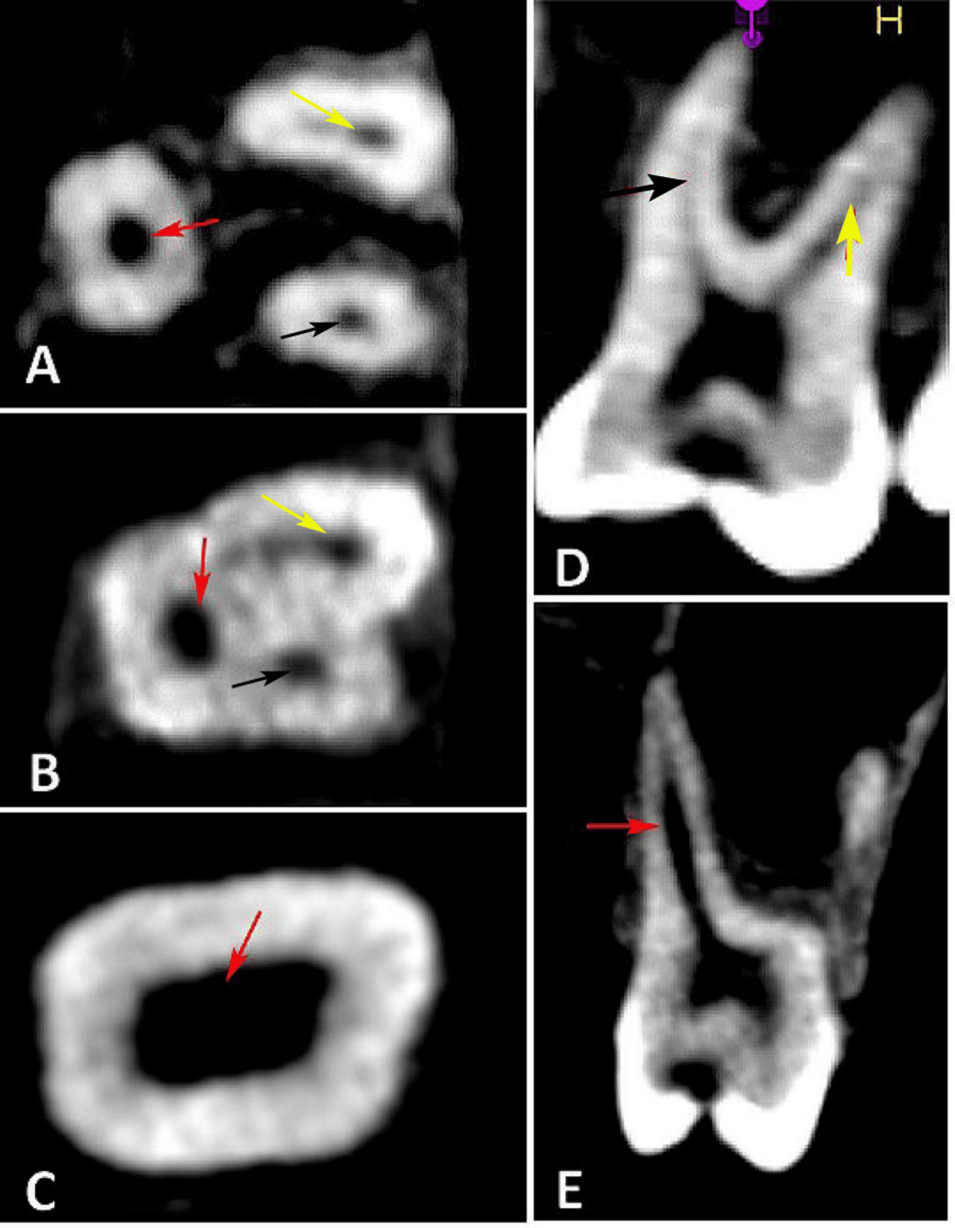
Root morphology of permanent maxillary first molar (A) root canals at the apical area showing the palatal canal (red arrow), mesiobuccal canal (black arrow), and distobuccal canal (yellow arrow); (B) root canals at the fulcrum area showing the palatal canal (red arrow), mesiobuccal canal (black arrow), and distobuccal canal (yellow arrow); (C) pulp chamber; (D) mesiobuccal (black arrow) and distobuccal canal (yellow arrow); and (E) palatal canal (red arrow).

Each maxillary first molar was examined to determine the number of roots, the number of canals within each root, and the canal configuration (Figure 1). Root canal morphology was initially classified according to the system proposed by Vertucci et al. [10] which categorizes canal configurations into eight types based on the number and course of canals from the pulp chamber to the apex: Type I (1-1), Type II (2-1), Type III (1-2-1), Type IV (2-2), Type V (1-2), Type VI (2-1-2), Type VII (1-2-1-2), and Type VIII (3-3). Morphologies that could not be classified using Vertucci’s system were further categorized according to the comprehensive coding system proposed by Ahmed et al. [11], which uses a structured O-C-F coding approach. In this system, the number of canal orifices, the canal pathway, and the number of apical foramina are encoded numerically (such as 2-1 indicates two orifices merging into one canal with a single foramen; 2-2 indicates two independent canals extending to two separate foramina). This system allows precise representation of complex canal configurations, including multiple divisions, re-merging patterns, and C-shaped morphologies, thereby overcoming the limitations of traditional classifications.

A pilot phase, involving 30 CBCT scans (excluded from the final sample), was conducted to standardize the evaluation protocol. Two experienced endodontists independently performed each scan. Interobserver reliability was measured using Cohen’s kappa statistic (target >0.85), and discrepancies were resolved by consensus discussion. Intraobserver reliability was confirmed by re-evaluation of 10% of the final sample after a two-week interval, yielding kappa values >0.90.

Statistical analysis

Data were analyzed using SPSS Statistics version 26 (IBM Corp., Armonk, NY, USA). Descriptive statistics, including frequencies and percentages, were calculated to describe the distribution of root and canal configurations in the permanent maxillary first molars according to different classification systems for MB, distobuccal, and palatal roots. Inter-rater and intra-rater reliability during image evaluation were assessed using Cohen’s kappa statistic in the pilot and final phases (values >0.85 and >0.90, respectively). Agreement between the two classification systems was evaluated using Cohen’s kappa coefficient, with the following interpretation: <0.00 = poor, 0.00-0.20 = slight, 0.21-0.40 = fair, 0.41-0.60 = moderate, 0.61-0.80 = substantial, 0.81-1.00 = almost perfect to perfect. The association between categorical variables was examined using the chi-squared test or Fisher’s exact test when the expected cell frequencies were low. Correspondence analysis was performed as an exploratory multivariate technique to visualize the relationships between Vertucci types (rows) and Ahmed codes (columns), with dimensions interpreted based on eigenvalue contributions and coordinate proximity. All tests were two-tailed, with a p-value <0.05 considered statistically significant.

## Results

A total of 300 permanent maxillary first molars from 300 individuals (mean age 28.45 ± 8.23 years) were evaluated using CBCT (Table 1). The MB root exhibited the greatest anatomical complexity, whereas the distobuccal and palatal roots showed predominantly simple canal configurations in both classification systems.

**Table 1:** Demographic characteristics of the study population (n = 300). n = number of participants; SD = standard deviation

Parameters		Unit	Value
Age	Years	Mean ± SD	28.45 ± 8.23
Sex	Male	N (%)	133 (44.3%)
	Female	N (%)	167 (55.7%)

In the MB root, the Vertucci et al. classification revealed a predominance of Types II and IV, followed by Type I, with smaller proportions of Type III and Type V, unclassifiable morphologies, and rare C-shaped forms (Table 2). The distobuccal and palatal roots were overwhelmingly Type I, with only minimal representation of other configurations and no unclassifiable or C-shaped cases.

**Table 2:** Distribution of root canal morphology based on Vertucci classification (n = 300). MB = mesiobuccal root; DB = distobuccal root; P = palatal root; n = number of teeth

Vertucci type	MB, n (%)	DB, n (%)	P, n (%)
Type I	72 (24.0%)	285 (95.0%)	284 (94.7%)
Type II	97 (32.3%)	12 (4.0%)	6 (2.0%)
Type III	18 (6.0%)	0 (0%)	0 (0%)
Type IV	84 (28.0%)	3 (1.0%)	8 (2.6%)
Type V	12 (4.0%)	0 (0%)	2 (0.7%)
Type VI	0 (0%)	0 (0%)	0 (0%)
Type VII	0 (0%)	0 (0%)	0 (0%)
Type VIII	0 (0%)	0 (0%)	0 (0%)
C‑shaped	2 (0.7%)	0 (0%)	0 (0%)
Unclassifiable	15 (5.0%)	0 (0%)	0 (0%)

The Ahmed et al. classification provided greater descriptive precision, particularly for the MB root, where the most frequent patterns corresponded to codes indicating division into two canals (primarily 2-1) and two orifices with a single apical foramen (primarily 2-2) (Table 3). Several less common but more intricate multi-division, re-merging, and continuous C-shaped morphologies were identified exclusively in MB roots. No cases remained unclassified using this system. Distobuccal and palatal roots showed a strong predominance of the single-canal code.

**Table 3:** Distribution of root canal morphology based on Ahmed classification (n = 300). MB = mesiobuccal root; DB = distobuccal root; P = palatal root; n = number of teeth

Ahmed code	MB, n (%)	DB, n (%)	P, n (%)
1	72 (24.0%)	285 (95.0%)	284 (94.7%)
2‑1	98 (32.6%)	9 (3.0%)	6 (2.0%)
1‑2‑1	15 (5.0%)	0 (0%)	0 (0%)
2‑2	86 (28.7%)	6 (2.0%)	8 (2.6%)
1‑2	12 (4.0%)	0 (0%)	2 (0.7%)
2-5-3-2	8 (2.7%)	0 (0%)	0 (0%)
3-2-1	5 1.6%)	0 (0%)	0 (0%)
1-2-1-2-1	2 (0.6%)	0 (0%)	0 (0%)
C (C‑shaped)	2 (0.6%)	0 (0%)	0 (0%)
Unclassified	0 (0%)	0 (0%)	0 (0%)

Cross-tabulation of the MB root demonstrated a strong overall correspondence between the Vertucci et al. and Ahmed et al. systems, with most Vertucci types mapping directly to the expected Ahmed codes and previously unclassifiable cases redistributed among several complex Ahmed configurations (Table 4).

**Table 4:** Cross-tabulation between Vertucci and Ahmed classifications in the mesiobuccal root (n = 300). Values represent frequency counts.

Vertucci/Ahmed	1	2-1	1-2-1	2-2	1-2	2-5-3-2	3-2-1	1-2-1-2-1	C-shaped	Unclassified	Total
Type I	70	0	0	2	0	0	0	0	0	0	72
Type II	0	95	0	1	1	0	0	0	0	0	97
Type III	0	3	15	0	0	0	0	0	0	0	18
Type IV	2	0	0	82	0	0	0	0	0	0	84
Type V	0	0	0	1	11	0	0	0	0	0	12
C-shaped	0	0	0	0	0	0	0	0	2	0	2
Unclassified	0	0	0	0	0	8	5	2	0	0	15
Total	72	98	15	86	12	8	5	2	2	0	300

Chi-square analysis confirmed a highly significant association between the two classification systems for all three root types (Table 5). Cohen’s kappa values indicated almost perfect agreement in the MB and distobuccal roots and perfect agreement in the palatal root, reflecting excellent concordance overall, with a slightly lower (although still very high) value in the MB root attributable to its greater anatomical variability (Table 6).

**Table 5:** Association between Vertucci and Ahmed classifications (chi-square test). *: P-values <0.05 are considered statistically significant. χ² = chi-square statistic; df = degrees of freedom; MB = mesiobuccal root; DB = distobuccal root; P = palatal root

Root	χ² value	df	P-value	Significance
MB	612.4	54	<0.001*	Highly significant
DB	520.7	4	<0.001*	Highly significant
P	480.2	9	<0.001*	Highly significant

**Table 6:** Agreement between Vertucci and Ahmed classifications (Cohen’s kappa analysis). *: P-values <0.05 are considered statistically significant. κ = Cohen’s kappa coefficient; MB = mesiobuccal root; DB = distobuccal root; P = palatal root

Root	Kappa (κ)	Standard error	P-value	Interpretation
MB	0.96	0.02	<0.001*	Almost perfect agreement
DB	0.98	0.01	<0.001*	Almost perfect agreement
P	1	0	<0.001*	Perfect agreement

**Figure 2:**
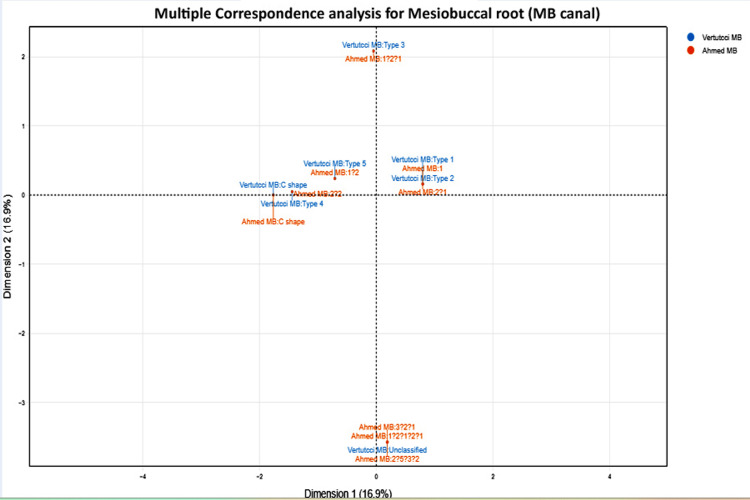
Multiple correspondence analysis (MCA) plot illustrating the association between mesiobuccal (MB) root canal morphology classifications by Vertucci and Ahmed classification. The plot displays two principal dimensions, with the percentage of total variance explained by each dimension indicated as Dimension 1: 16.9%, Dimension 2: 18.9%. Two main clusters are evident: the first cluster (right side) corresponds to Type 1 (Vertucci) and “1” (Ahmed), representing strong agreement for the simplest canal configuration; the second cluster (left and center) comprises Types 3, 4, 5 (Vertucci) and “C shape” (Ahmed), reflecting more complex morphologies where interobserver agreement is more variable. The proximity of points indicates similar classification patterns between observers.

Correspondence analysis visually confirmed these relationships, showing tight clustering of the corresponding Vertucci types and Ahmed codes along the primary dimension (increasing canal complexity), while rare and highly variant morphologies appeared as distinct outliers (Figure 2). This pattern further highlights the enhanced ability of the Ahmed classification to discriminate complex anatomies. No statistically significant differences in root or canal configuration distributions were observed between males and females (p > 0.05). These region-specific observations indicate substantial anatomical complexity in the MB root of permanent maxillary first molars in the Meerut population, with the majority of teeth presenting configurations that include the second MB canal system. These findings emphasize the clinical importance of anticipating such variability in endodontic access cavity design, canal location, instrumentation, and obturation.

## Discussion

The present in vivo CBCT investigation in the Meerut population of northern India revealed substantial anatomical complexity confined primarily to the MB root of permanent maxillary first molars, where Vertucci Types II and IV predominated, collectively indicating an MB2 canal system in approximately 60% of the cases. This prevalence closely parallels findings from other Indian CBCT studies, including 61.9% reported in a Karnataka subpopulation and 77.5% documented in a dedicated North Indian cohort [8,12]. Slightly higher rates have been observed in broader Indian samples, while southern Indian investigations have ranged between 36% and 72% [13,14]. Such consistency across Indian subpopulations underscores a characteristic ethnic pattern likely shaped by shared genetic and migratory influences on the subcontinent.

Internationally, the observed MB2 frequency aligns with pooled estimates from large-scale CBCT meta-analyses, which reported a global prevalence of 48.0% in Venezuela and 97.6% in Belgium [15]. For instance, a recent systematic review of the Han Chinese population reported a prevalence of 63.7%, while worldwide analyses confirmed similar regional variability [16]. These differences highlight the role of ethnic, racial, and environmental factors in odontogenesis, reinforcing that population-specific data are indispensable for accurate preoperative planning rather than relying on universal textbook averages derived from clearing techniques performed decades ago.

A key strength of the present study is the parallel application of Vertucci’s and Ahmed et al.’s classification systems. Although Vertucci’s eight-type system captured the majority of configurations, 5% of MB roots remained unclassifiable, underscoring its limitations when dealing with intricate multi-division and re-merging patterns. In contrast, the Ahmed coding system (O-C-F) successfully classified each specimen, revealing additional complex morphologies exclusively in the MB root. A multinational CBCT study by Pertek Hatipoğlu et al. [17] across 22 countries similarly demonstrated the superior comprehensiveness of the Ahmed et al. classification system over Vertucci’s in describing root and canal morphology, as it successfully categorized all examined maxillary first premolars without leaving any unclassified cases, whereas Vertucci’s system failed to classify 1.8-2.7% of specimens, paralleling the present findings in maxillary first molars where 5% of MB roots remained unclassifiable under Vertucci but were fully accommodated by Ahmed coding. The almost perfect to perfect Cohen’s kappa values obtained in our study further validate the complementary nature of the two systems, with the Ahmed classification system offering enhanced clinical utility for documentation, communication, and research.

Correspondence analysis provided a clear visual map of the strong concordance between the corresponding Vertucci types and Ahmed codes, with simple single-canal patterns clustering tightly and complex variants positioned as outliers along the primary dimension of canal complexity. This multivariate approach adds interpretive depth and may prove valuable in future meta-analyses exploring classification agreement across populations.

Similar advantages of the Ahmed classification were reported by Saber et al. [18] in a CBCT study of maxillary premolars in an Egyptian subpopulation, where the system fully classified all root and canal configurations without leaving any unclassified cases, providing greater precision for complex variants than Vertucci’s system. This was echoed by Alsayed Tolibah et al. [19] in an epidemiological CBCT analysis of extracted mandibular premolars from adolescent patients in Damascus, where all specimens were classifiable under Ahmed’s O-C-F coding, whereas Vertucci’s classification left certain intricate morphologies unaddressed. These observations parallel the present findings in permanent maxillary first molars in the Meerut population, where 5% of MB roots remained unclassifiable under Vertucci, but were precisely accommodated and described in detail by the Ahmed system, highlighting its superior granularity for complex canal patterns across different tooth types and geographic populations.

The absence of statistically significant sex-based differences in the root and canal configurations was consistent with previous studies [13,16,20]. The lack of sex dimorphism in the present Meerut sample may reflect regional genetic homogeneity or balanced demographic distribution. Overall, the findings confirm that the MB root remains the primary site of anatomical challenge in maxillary first molars among individuals from northern India, with the distobuccal and palatal roots exhibiting a reassuringly simple Type I/code 1 morphology in over 94% of cases. These region-specific data fill an important gap, as prior Indian CBCT research has predominantly originated from southern or western states, and northern urban populations, such as Meerut, subject to diverse migratory influences, have not been systematically mapped. However, as the present sample was derived from clinically indicated CBCT scans rather than a true screening population, the reported prevalence of canal configurations may differ from that observed in population-based screening studies.

Clinical implications and limitations

Clinically, these results mandate heightened vigilance during the endodontic management of maxillary first molars in the Meerut region and comparable northern Indian populations. Preoperative CBCT, when indicated, should be routinely scrutinized for MB2 canals, guiding conservative access design, ultrasonic troughing 1.5-2.5 mm palatal to the main MB orifice, and the use of enhanced magnification to prevent missed canals, the leading cause of treatment failure. The adoption of the Ahmed classification in clinical records and referral communications will facilitate more precise treatment planning and interdisciplinary dialogue.

The limitations of the study include its single-center design at one teaching hospital, potential selection bias toward patients already requiring CBCT for other diagnostic purposes, exclusion of teeth with severe calcification or incomplete roots, and the use of standard-resolution imaging (voxel ≤0.2 mm) rather than ultra-high-resolution protocols. Age-stratified subgroup analyses were not performed, and generalizability beyond the 18-70-year age range to rural populations remains to be established. Additionally, the use of CBCT scans obtained for specific clinical indications may introduce spectrum bias, potentially influencing the observed prevalence of canal configurations and limiting generalizability. The absence of age-stratified analysis restricts the evaluation of age-related morphological variations, particularly those associated with secondary dentin deposition and canal calcification. Multicenter studies incorporating micro-CT validation and broader demographic representations are recommended to further strengthen these observations.

## Conclusions

This in vivo CBCT study of 300 permanent maxillary first molars in the Meerut population revealed substantial anatomical complexity, predominantly in the MB root, with approximately 60% exhibiting configurations indicative of an MB2 canal system. The Ahmed classification demonstrated superior descriptive capability by fully categorizing all morphologies, including the 5% of MB roots unclassifiable under Vertucci, while maintaining an almost perfect concordance with the traditional system. No significant sex-based differences were observed between groups. These region-specific findings emphasize the clinical necessity of anticipating MB2 canals during the endodontic treatment of maxillary first molars in northern Indian populations, advocating routine preoperative CBCT scrutiny, enhanced magnification, and adoption of the Ahmed classification for precise documentation and improved treatment predictability.
